# Improving l-serine formation by *Escherichia coli* by reduced uptake of produced l-serine

**DOI:** 10.1186/s12934-020-01323-2

**Published:** 2020-03-14

**Authors:** Chenyang Wang, Junjun Wu, Binchao Shi, Jiping Shi, Zhijun Zhao

**Affiliations:** 1 Biorefinery Laboratory, Shanghai Advanced Research Institute, Chinese Academy of Sciences, 99 Haike Road, Shanghai, 201210 China; 2 University of Chinese Academy of Sciences, 19 Yuquan Road, Beijing, 100049 China; 3 College of Food Science and Technology, Nanjing Agricultural University, 1 Weigang Road, Nanjing, 210095 China; 4 College of Life Science, Shihezi University, 221 Beisi Road, Shihezi, 832003 China; 5 School of Life Science and Technology, ShanghaiTech University, Shanghai, 201210 China

**Keywords:** l-serine, Uptake system, Gene knockout, Fermentation of l-serine

## Abstract

**Background:**

Microbial de novo production of l-serine, which is widely used in a range of cosmetic and pharmaceutical products, has attracted increasing attention due to its environmentally friendly characteristics. Previous pioneering work mainly focused on l-serine anabolism; however, in this study, it was found that l-serine could be reimported through the l-serine uptake system, thus hampering l-serine production.

**Result:**

To address this challenge, engineering via deletion of four genes, namely, *sdaC*, *cycA*, *sstT* and *tdcC*, which have been reported to be involved in l-serine uptake in *Escherichia coli*, was first carried out in the l-serine producer *E. coli* ES. Additionally, the effects of these genes on l-serine uptake activity and l-serine production were investigated. The data revealed an abnormal phenomenon regarding serine uptake activity. The serine uptake activity of the Δ*sdaC* mutant was 0.798 nmol min^−1^ (mg dry weight) ^−1^ after 30 min, decreasing by 23.34% compared to that of the control strain. However, the serine uptake activity of the single *sstT*, *cycA* and *tdcC* mutants increased by 34.29%, 78.29% and 48.03%, respectively, compared to that of the control strain. This finding may be the result of the increased level of *sdaC* expression in these mutants. In addition, multigene-deletion strains were constructed based on an *sdaC* knockout mutant. The Δ*sdaC*Δ*sstT*Δ*tdcC* mutant strain exhibited 0.253 nmol min^−1^ (mg dry weight) ^−1^l-serine uptake activity and the highest production titer of 445 mg/L in shake flask fermentation, which was more than three-fold the 129 mg/L production observed for the parent. Furthermore, the Δ*sdaC*Δ*sstT*Δ*tdcC* mutant accumulated 34.8 g/L l-serine with a yield of 32% from glucose in a 5-L fermenter after 36 h.

**Conclusion:**

The results indicated that reuptake of l-serine impairs its production and that an engineered cell with reduced uptake can address this problem and improve the production of l-serine in *E. coli*.

## Background

l-Serine is a vital component of metabolism and an important material used in the pharmaceutical and cosmetic industries, with a 5–7% annual growth rate in its market demand currently [1, 2]. The direct fermentation of cheaper carbon sources to obtain l-serine has become a promising production method because this method is environmentally friendly and allows easy extraction [3].

**Fig. 1:**
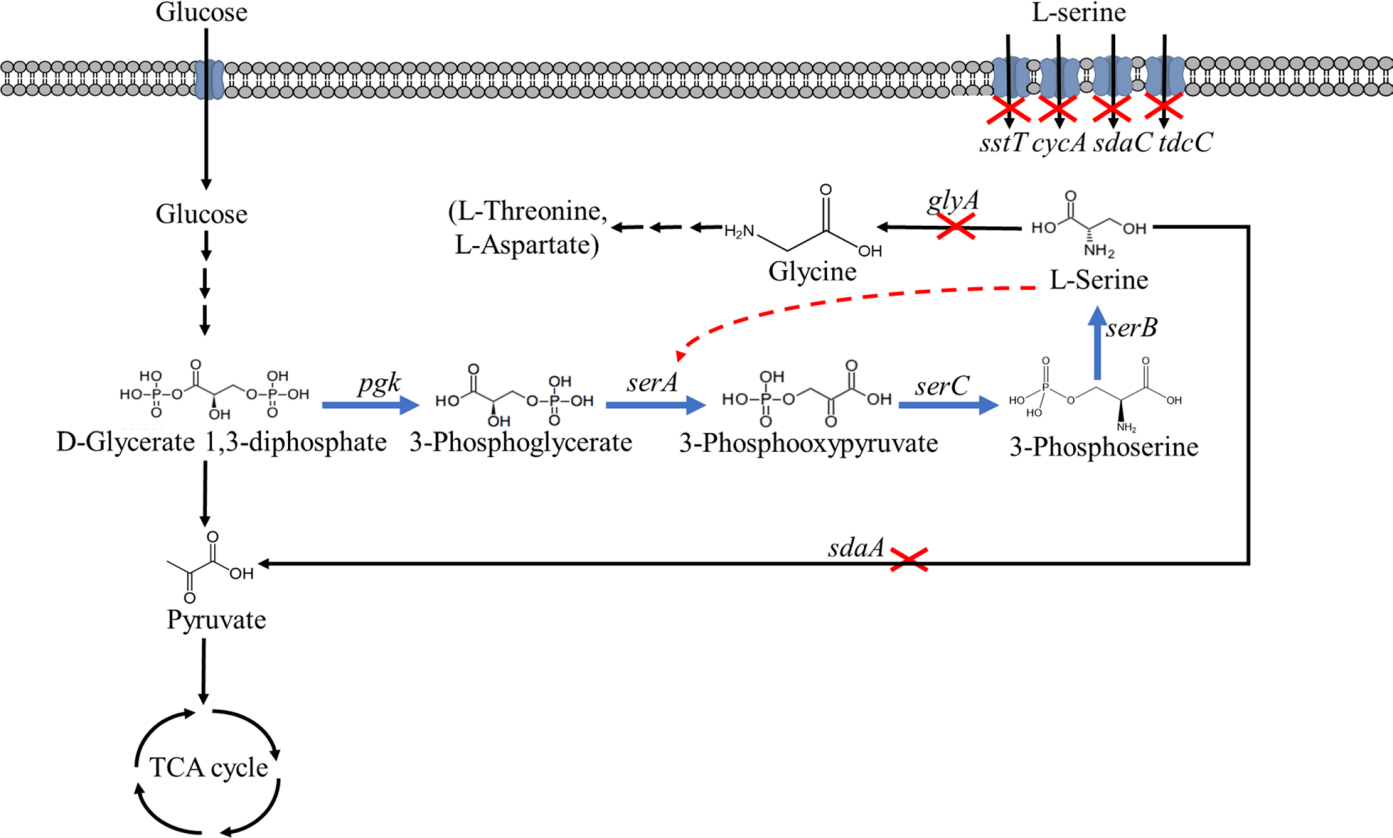
Biosynthesis pathways and uptake systems of l-serine in *E. coli*. Blue arrows indicate overexpression of the relevant genes. Red “X”s indicate deletion of relevant genes. The red dashed line indicates feedback inhibition

Numerous exciting studies have demonstrated the successful microbial production of l-serine. For example, Peters-Wendisch al. [4] constructed a *Corynebacterium glutamicum* strain by examining key genes; overexpressing *serA*^*fr*^ (fr, feedback inhibition resistance), *serB,* and *serC*; and deleting *sdaA*. Subsequently, production of 36 g/L l-serine was achieved by controlling SHMT activity with a folate supply in a 60-h fed-batch fermentation process [5]. Zhu et al. [6] also obtained a *C. glutamicum* strain engineered to minimize the byproducts l-alanine and l-valine by deleting *alaT*, *avtA* and *ilvN* and achieved l-serine production of 42.6 g/L in a 96-h fed-batch fermentation process. In addition, *Escherichia coli* has been widely engineered for l-serine production due to its easy genetic manipulation and short growth period [7–9] (Fig. 1). For instance, *E. coli* DH5ɑ was engineered to enhance l-serine precursor production and strengthen the l-serine synthesis pathway by overexpressing *serA*^*fr*^*, serB* and *serC*. The recombinant strain produced 8.34 g/L l-serine from glucose [10]. In 2016, Mundhada et al. [11] developed a strain of *E. coli* MG1655 lacking the l-serine degradation genes, *sdaA*, *sdaB* and *tdcG*, and the l-serine hydroxymethyltransferase gene *glyA* and overexpressing the l-serine synthesis genes *serA*^*fr*^, *serB* and *serC* and the cysteine/homoserine transporter *eamA*, which led to l-serine production of 11.7 g/L. Furthermore, the strain was modified for improved l-serine tolerance by adaptive laboratory evolution, and the l-serine production increased to 37 g/L after 52 h of fermentation [12].

Notably, the previous study mainly focused on enhancing l-serine biosynthesis and decreasing the degradation of this compound. However, external l-serine could be reimported into cells and used for protein synthesis and to increase cell viability. This phenomenon may inhibit l-serine production during fermentation. Blocking reuptake by modifying the uptake process has been proven to be an efficient method. For instance, deletion of the l-tryptophan uptake gene *mtr* resulted in a decrease in l-tryptophan uptake by 48% in *E. coli* [13], deletion of the l-threonine uptake gene *sstT* reduced l-threonine uptake activity by 50% in *E. coli* [14], and mutation of the aminobutyric acid (GABA) uptake gene GabP_Cg_ caused the strain to lose the ability to take up any GABA [15]. However, thus far, no study has investigated the effects of the l-serine uptake system on l-serine production and growth in *E. coli*.

Previously, four genes, namely, *sstT*, *cycA*, *sdaC* and *tdcC*, were reported to be related to l-serine uptake in *E. coli* [16–23]. In the present study, first, the occurrence of l-serine reuptake was clearly demonstrated, and the functions of these four genes were verified by overexpression of these genes in the l-serine-producing strain ES. Furthermore, the four genes were knocked out in combination in ES. Single‐gene deletion mutants and multi‐gene deletion mutants were generated, and their growth, l-serine uptake activity and l-serine production were evaluated.

## Results

### Investigating whether l-serine could be reimported by *E. coli*

**Fig. 2:**
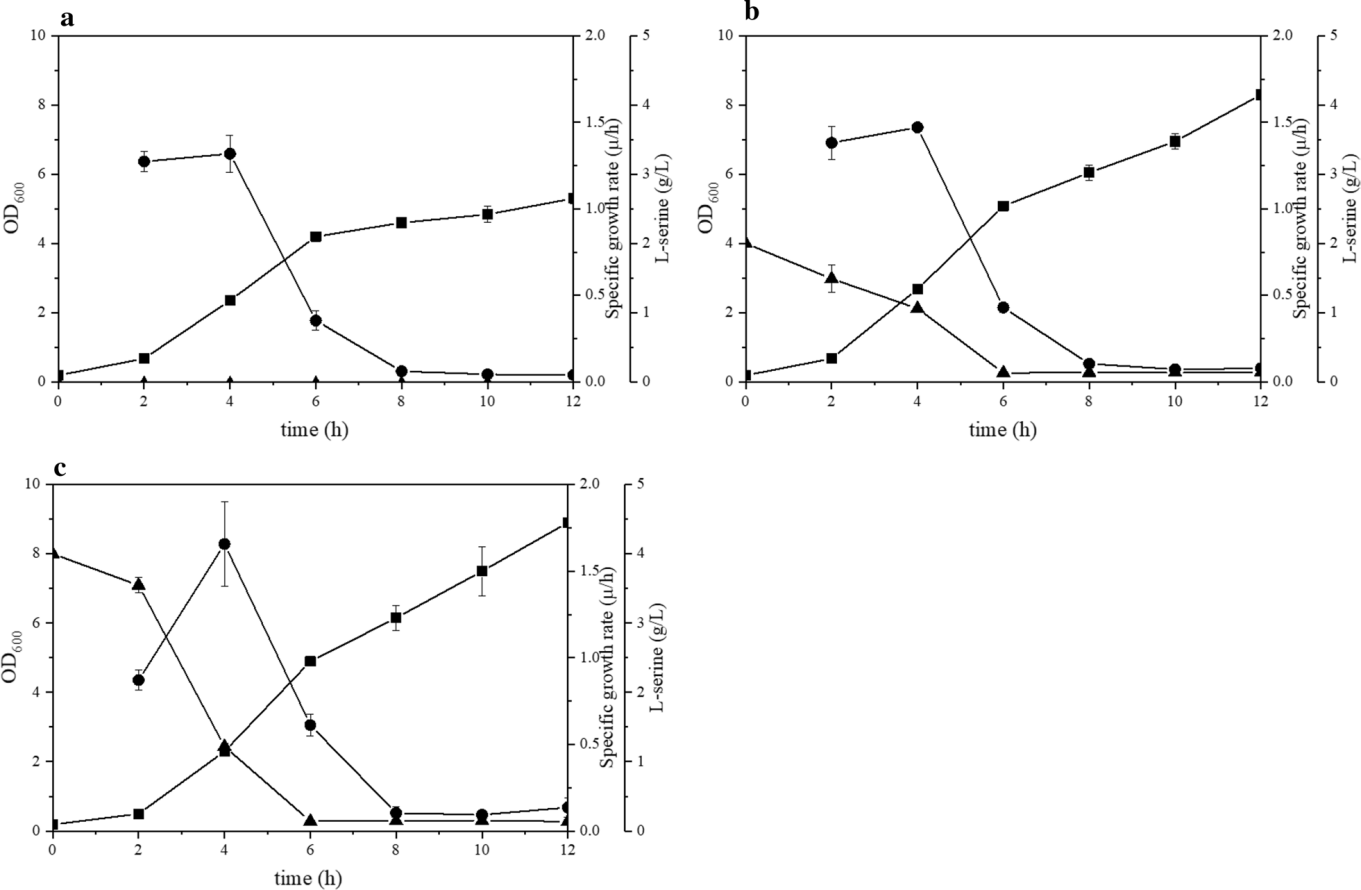
Time courses of l-serine concentration and biomass. l-serine concentration (filled triangle), cell growth (filled square) and specific growth rate (filled circle) of *E. coli* ES growing in LB medium without l-serine (**a**), with 2 g/L l-serine (**b**) and with 4 g/L l-serine (**c**)

To determine whether l-serine was reimported, *E. coli* ES was inoculated into Luria-Bertani (LB) medium with an additional 2 g/L or 4 g/L l-serine. An HPLC chromatogram of the l-serine standard solution is shown in Additional file 1: Fig. S1. As shown in Fig. 2, the l-serine concentration decreased to 1.05 g/L and 1.17 g/L at 4 h. Then, the l-serine concentrations were further reduced to less than 0.15 g/L at 6 h. It was observed that the maximum specific growth rates of the strains with an additional 2 g/L and 4 g/L l-serine were 1.47 h^−1^ and 1.65 h^−1^, respectively, which were 1.12- and 1.26-fold that of the corresponding control strain, respectively. The final optical densities at 600 nm (OD_600_) of the strains in LB medium with an additional 2 g/L and 4 g/L l-serine increased by 56% and 67%, respectively, compared to that of the control (OD_600_ ~ 5.3). This demonstrated that l-serine could be imported and metabolized and provided easily assimilable carbon and nitrogen sources for cell growth. This raises the question of how the uptake takes place and whether it has any effect on l-serine accumulation.

### Relevance of transporters for l-serine uptake

**Fig. 3:**
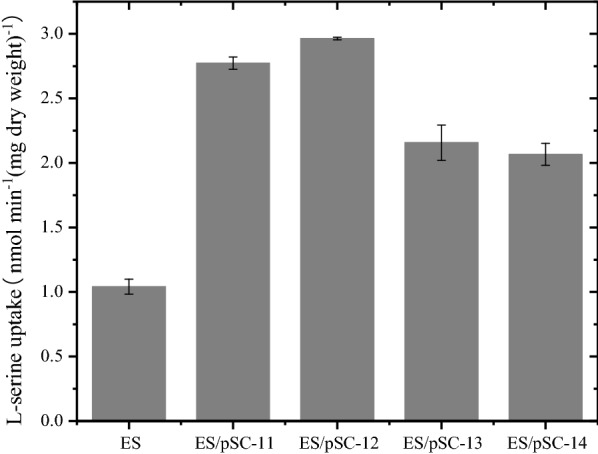
l-serine influx was assayed in *E. coli* ES and mutants overexpressing the l-serine uptake genes. The data represent the means ± SDs from three measurements. ES represents the parental strain

The four genes *sdaC*, *cycA*, *sstT* and *tdcC*, which were reported to be related to l-serine uptake, were overexpressed in *E. coli* ES [16–23]. The l-serine uptake activity of these strains was evaluated. As shown in Fig. 3, the l-serine uptake activity (30 min) of ES/pSC-12, ES/pSC-11, ES/pSC-14 and ES/pSC-13 was 2.983, 2.79, 2.3 and 2.166 nmol min^−1^ (mg dry weight)^−1^, respectively, increasing by 186%, 168%, 121% and 108% compared to that of the parent strain of ES (1.04 nmol min^−1^ (mg dry weight)^−1^). The time courses of l-serine uptake are shown in Additional file 2: Fig. S2. This result indicated that *cycA* and *sda*C played critical roles and that *sstT* and *tdcC* were also important in l-serine uptake activity.

### Effect of single-gene deletions on l-serine uptake

**Fig. 4:**
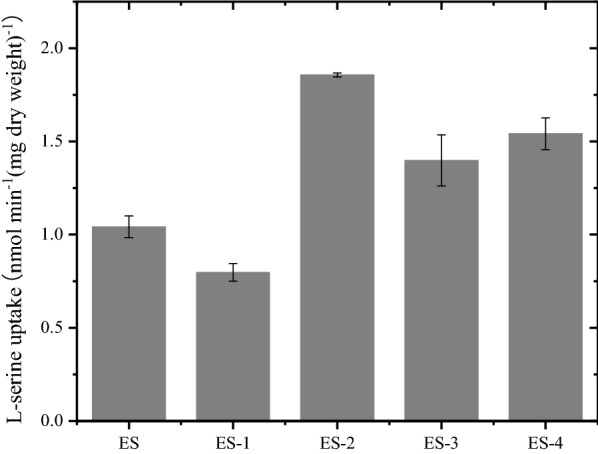
l-serine influx was assayed in *E. coli* ES and single-gene knockout mutants. The data represent the means ± SDs from three measurements. ES represents the parental strain. ES-1: ES Δ*sdaC*; ES-2: ES Δ*cycA*; ES-3: ES Δ*sstT*; ES-4: ES Δ*tdcC*

**Fig. 5:**
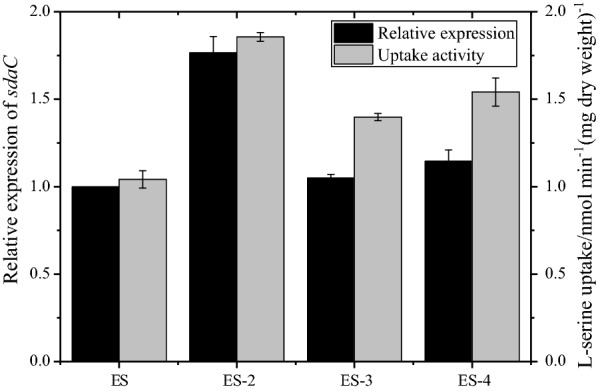
Relative expression of *sdaC* in *E. coli* ES and single-gene knockout mutants. ES represents the parental strain, and the relative gene expression in ES was onefold. ES-2: ES Δ*cycA*; ES-3: ES Δ*sstT*; ES-4: ES Δ*tdcC*

The four genes, *sdaC*, *cycA*, *sstT* and *tdcC*, were knocked out, resulting in strains ES-1, ES-2, ES-3 and ES-4. As shown in Fig. 4 and Additional file 2: Fig. S3, the l-serine uptake activity (30 min) of ES-1 was 0.79 nmol min^−1^ (mg dry weight)^−1^, decreasing by 23% compared to that of the parent strain of ES (1.04 nmol min^−1^ (mg dry weight)^−1^). It was surprising that the l-serine uptake activity of ES-2, ES-4 and ES-3 increased by 77%, 48% and 33% compared to that of the control. This abnormal phenomenon was explored through a series of real-time quantitative reverse‐transcription PCR (RT-qPCR) experiments. As shown in Fig. 5, deletion of the *cycA* and *tdcC* genes led to a 1.76- and 1.15-fold increase in the relative expression of *sdaC*, respectively, which was consistent with the increase in l-serine uptake activity in the two mutants. However, the *sstT* mutants showed similar expression of *sdaC* with increased l-serine uptake activity compared to ES. These results illustrated that certain regulatory mechanisms of the l-serine uptake system remain unknown.

### Effect of multigene deletions on l-serine uptake

**Fig. 6:**
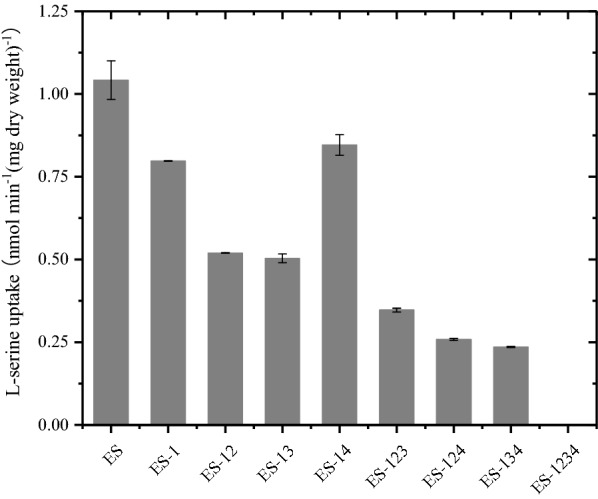
l-serine influx was assayed in *E. coli* ES and multigene deletion mutants. The data represent the means ± SDs from three measurements. ES represents the parental strain. ES-1: ES Δ*sdaC*; ES-12: ESΔ*sdaC*Δ*cycA*; ES-13: ESΔ*sdaC*Δ*sstT;* ES-14: ESΔ*sdaC*Δ*tdcC*; ES-123: ES Δ*sdaC*Δ*cycA*Δ*sstT*, ES-124: ESΔ*sdaC*Δ*cycA*Δ*tdcC*; ES-134: ESΔ*sdaC*Δ*sstT*Δ*tdcC;* ES-1234: ESΔ*sdaC*Δ*cycA*Δ*sstT*Δ*tdcC*

The above results demonstrated that *sdaC* played a significant role in the l-serine uptake system, but the roles of *cycA, sstT* and *tdcC* in the uptake system remained unclear. Therefore, the three genes were knocked out combinatorially in the single-gene-deletion strain ES-1, resulting in seven multigene-deletion strains. The mutant strains were named ES-12 (ES *ΔsdaCΔcycA*), ES-13 (ES *ΔsdaCΔsstT*), ES-14 (ES *ΔsdaCΔtdcC*), ES-123 (ES *ΔsdaCΔcycAΔsstT*), ES-124 (ES*ΔsdaCΔcycAΔtdcC*), ES-134 (ES *ΔsdaCΔsstTΔtdcC*) and ES-1234 (ES *ΔsdaCΔcycAΔsstTΔtdcC*). As shown in Fig. 6 and Additional file 2: Fig. S4, among the double-gene-deletion strains, both strains ES-12 and ES-13 showed a low l-serine uptake activity of nearly 0.50 nmol min^−1^ (mg dry weight)^−1^, decreasing by 52% compared to that of ES. However, another double-gene deletion strain, ES-14, showed 0.846 nmol min^−1^ (mg dry weight)^−1^l-serine uptake activity, which was similar to that of ES-1. Among the triple-gene deletion mutants, the l-serine uptake activity of ES-123 was 0.347 nmol min^−1^ (mg dry weight)^−1^. ES-124 and ES-134 showed an l-serine uptake activity of nearly 0.24 nmol min^−1^ (mg dry weight)^−1^, decreasing by 76% compared to that of ES. Finally, when all four genes were deleted (ES-1234), the l-serine uptake activity decreased to near zero, which implied that the strain could not import extracellular l-serine effectively.

### The impact of the deletion of l-serine uptake genes on growth and l-serine production in shake flask fermentation

**Fig. 7:**
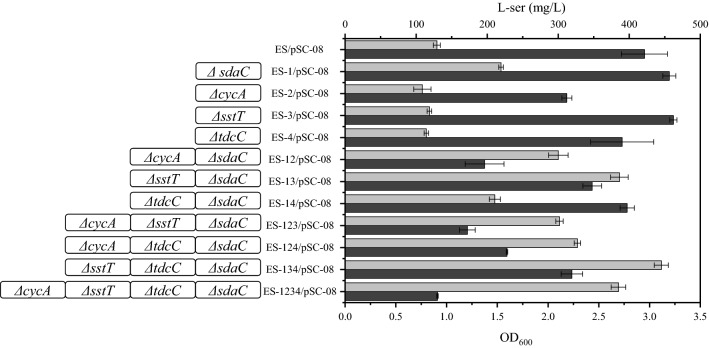
Biomass and l-serine production of different strains in shake flask fermentations. The data represent the means ± SDs from three measurements. The genotype of the strains and the biomass (dark gray) and L-serine production (light gray) of different strains are shown

To evaluate the l-serine production capability of the l-serine uptake system mutants, the mutant strains were transformed with the plasmid pSC-08 containing the l-serine synthesis genes *serA*^*fr*^, *serB* and *serC* and the 3-phosphoglycerate kinase gene *pgk* and shake flask fermentation was performed. Among single-gene deletion mutants, deletion of *sdaC*, *sstT* and *tdcC* had a slight effect on the density of the cell cultures (Fig. 7), including that all strains in which *cycA* was deleted showed poor growth, and ES-2/pSC-08 showed a low final OD_600_ of 2.19, which was 30% less than that of ES/pSC-08 (OD_600_ ~ 2.9). The strains exhibited poor growth when more genes were knocked out. For example, the final OD_600_ values of ES-1/pSC-08, ES-13/pSC-08 and ES-134/pSC-08 were 3.2-, 2.43- and 2.23, respectively, which were 1.1-, 0.82- and 0.77-fold the value for ES/pSC-08. respectively.

In addition, as shown in Fig. 7, the strains with lower l-serine uptake activity exhibited higher l-serine production. For instance, ES-1/pSC-08, ES-13/pSC-08 and ES-134/pSC-08 showed 23%, 48% and 77% lower l-serine uptake activity and 69%, 196% and 242% higher l-serine production, respectively, than the control strain ES/pSC-08 (1.04 nmol min^−1^ (mg dry weight)^−1^, 130 mg/L). ES-134/pSC-08 achieved the highest l-serine production (445 mg/L) in shake flask fermentation. However, ES-1234/pSC-08, which had nearly no l-serine uptake activity, produced only 384 mg/L l-serine and showed the poorest cell growth.

### Fed-batch fermentation in a 5-L fermenter

**Fig. 8:**
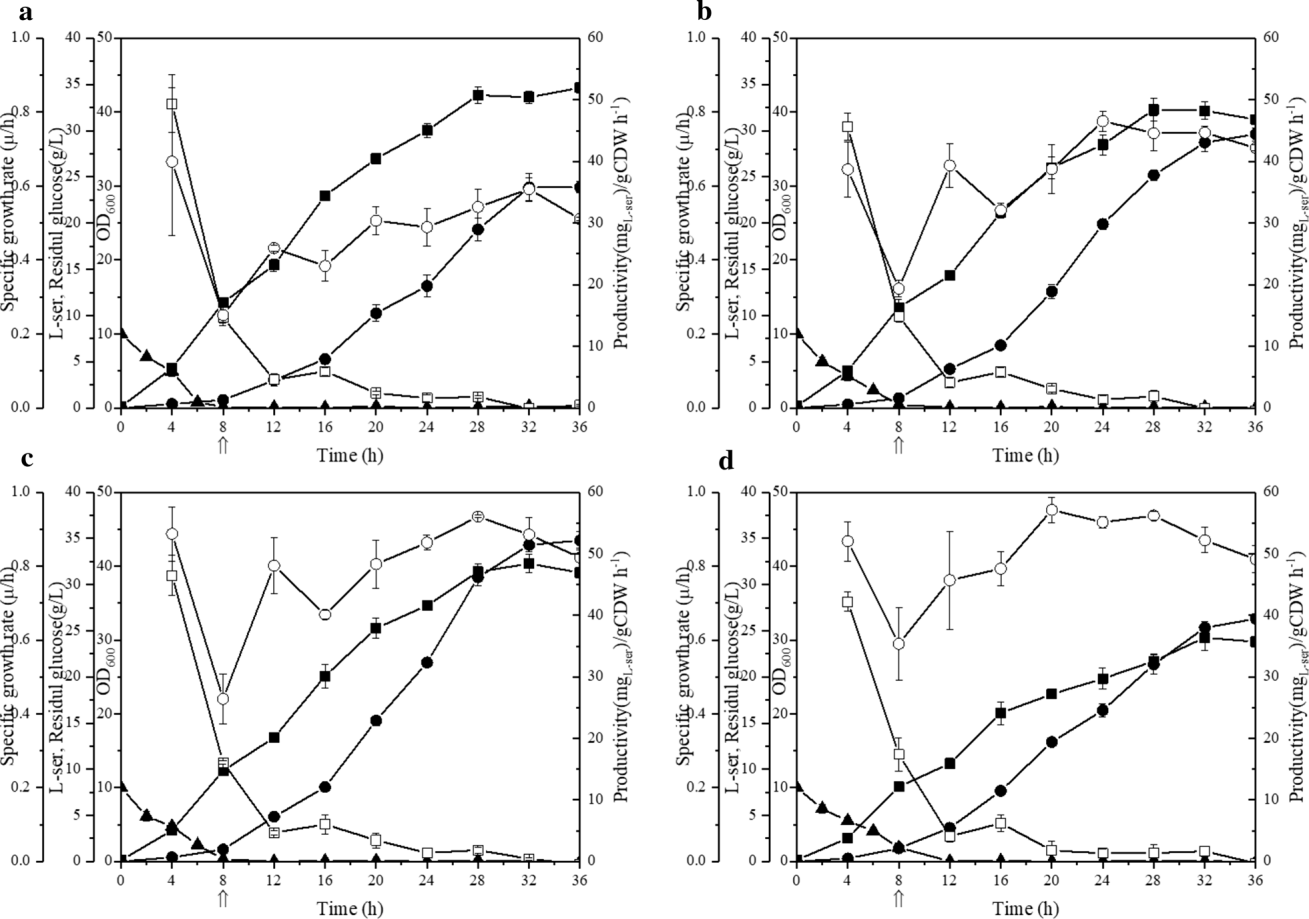
Fed-batch fermentations of ES-1/pSC-08, ES-13/pSC-08, ES-134/pSC-08 and ES-1234/pSC-08. ES-1/pSC-08 (**a**), ES-13/pSC-08 (**b**), ES-134/pSC-08 (**c**), ES-1234/pSC-08 (**d**). Cell growth (filled square), l-serine concentration (filled circle), residual glucose concentration (up filled triangle), specific growth rate (open square), and l-serine productivity (open circle) are shown; the arrow at 8 h indicates the starting point of induction

The four high-yield strains in shake flask fermentation, namely, ES-1/pSC-08, ES-13/pSC-08, ES-134/pSC-08 and ES-1234/pSC-08, were selected for a 36-h fed-batch fermentation in a 5-L fermenter. As shown in Fig. 8 and Additional file 3: Table S1, ES-1/pSC-08 produced 23.8 g/L l-serine with a yield of 0.25 g l-serine/g glucose. The production of ES-13/pSC-08 further increased to 29.6 g/L with a yield of 0.31 g l-serine/g glucose. As expected, the triple-deletion mutant strain ES-134/pSC-08 showed the highest production of 34.8 g/L with a yield of 0.32 g l-serine/g glucose, increasing by 46% compared to that of ES-1/pSC-08. The concentration of l-serine detected at the end of the ES-1234/pSC-08 fed-batch culture was 26.3 g/L, which was similar to that of ES-1/pSC-08, due to the lowest biomass (OD_600_ ~ 30) of ES-1234/pSC-08.

During the effective l-serine production time (12–36 h), the productivity of ES-1/pSC-08, ES-13/pSC-08, ES-134/pSC-08 and ES-134/pSC-08 was in the range of 23–36, 31–47, 40–56 and 46–57 mg l-serine/g CDW h^−1^, respectively (Fig. 8). Additionally, ES-134/pSC-08 achieved the highest l-serine titer. However, the l-serine production of ES-1234/pSC-08 was affected by its lowest OD_600_ although it showed the highest l-serine productivity.

## Discussion

l-Serine is an important biochemical building block, and the microbial production of l-serine from glucose provides a cost-competitive method. Previous studies focused on enhancing the metabolic flux of l-serine and inhibiting l-serine degradation. However, in this study, it was found that external l-serine was reimported into the *E. coli*, and nearly 4 g/L l-serine could be consumed at 6 h of cultivation. This may hinder high production of the l-serine-producing strain. The l-serine uptake system was deleted to prevent this phenomenon.

Four genes, *sdaC*, *cycA*, *sstT* and *tdcC*, were reported to be related to l-serine uptake. Only *sdaC* has been described as a highly specific l-serine transporter and belongs to the H^+^-symporter family [16, 17]. In this study, deletion of *sdaC* resulted in a 23% decrease in l-serine uptake (Fig. 4). The abnormally increasing influx of l-serine was attributed to the elevated expression of *sdaC* in the other single-gene mutations. *cycA*, which also belongs to the H^+^-symporter family, is the main alanine carrier and participates in l-serine and glycine uptake simultaneously in *E. coli* [18–20]. Among the single-gene mutations, only the *cycA* mutation led to a decline in the final OD_600_ of the cell culture. This may be due to the defective uptake of alanine when the transporter of alanine, *cycA*, was deleted, thus affecting cell growth. *sstT* is a Na^+^-dependent carrier and mediates the uptake of l-threonine and l-serine [17, 21]. Notably, t*dcC* is an H^+^-dependent threonine transporter and is involved in l-serine transport under anaerobic conditions [22, 23]. Nevertheless, in this study, l-serine uptake activity increased 108% in shake flask fermentation when *tdcC* was overexpressed with the promoter PR under aerobic conditions. The related regulatory mechanisms need further experiments to explore. These data indicated that *sdaC* was the most critical gene of the l-serine uptake system and that there was coordinated regulation among various l-serine-related genes.

This study provides evidence that low l-serine uptake activity contributes to high l-serine production, and similar gains have been achieved in other amino acid productions processes according to previous studies. For example, the l-threonine uptake rates of strains VL334 pYN7, ATCC 98082 pYN7 and BKIIMB-3996 pVIC40 were 4.7, 2.8 and 1.9 nmol min^−1^ (mg dry weight)^−1^, respectively, producing 9, 20 and 80 g/L l-threonine [14]. In this study, the strains ES-1 and ES-134 showed decreases in l-serine uptake activity of 30% and 75%, respectively, and increases in l-serine production of 67% and 245%, respectively, in shake flask fermentations (Figs. 6 and 7). The production of ES-134 was further increased to 34.8 g/L with a yield of 0.32 g l-serine/g glucose during a 36-h fed-batch bioconversion (Fig. 8). Notably, cell growth was affected when the l-serine uptake genes were deleted, and the production was subsequently indirectly impacted. For instance, ES-1234, which could barely import extracellular l-serine, only produced 26.3 g/L l-serine as a result of its poor growth. Therefore, the growth of the strain is an important factor during fermentation engineering.

## Conclusion

Deletion of the l-serine uptake system led to significant enhancement of l-serine production. The reduction of l-serine uptake activity could benefit l-serine production. The data show that *sdaC* is a key gene in the l-serine uptake system and that deletion of *sdaC* is important for improving l-serine production. Our studies have demonstrated that modification of the l-serine uptake system may be a useful strategy for improving l-serine production.

## Materials and methods

### Bacterial strains and plasmids

The *E. coli* ES strain is a laboratory stock that is a *sdaA* and *glyA* double-gene knockout mutant derived from wild-type *E. c*oli W3110. In the present study, four l-serine uptake genes, namely, *sdaC*, *cycA*, *sstT* and *tdcC*, were knocked out individually or combinatorially from the genome of *E. coli* ES. The primers shown in Additional file 4: Table S2, were used to conduct single- or multigene knockout mutations based on a previously reported method [24]. The strains constructed in this study were verified by sequencing. All strains are listed in Table 1.

**Table 1:** Strains used in this study

Strain	Description	Source or reference
W3110	K-12 wild-type (ATCC 27325)	Genetimes ExCell Technology, Inc
ES	W3110 (Δ*sdaA*, Δ*glyA*)	Stock in lab
ES-1	ES (Δ*sdaC*)	This work
ES-2	ES (Δ*cycA*)	This work
ES-3	ES (Δ*sstT*)	This work
ES-4	ES (Δ*tdcC*)	This work
ES-12	ES (Δ*sdaC*, Δ*cycA*)	This work
ES-13	ES (Δ*sdaC*, Δ*sstT*)	This work
ES-14	ES (Δ*sdaC*, Δ*tdcC*)	This work
ES-123	ES (Δ*sdaC*, Δ*cycA*, Δ*sstT*)	This work
ES-124	ES (Δ*sdaC*, Δ*cycA*, Δ*tdcC*)	This work
ES-134	ES (Δ*sdaC*, Δ*sstT*, Δ*tdcC*)	This work
ES-1234	ES (Δ*sdaC*, Δ*cycA*, Δ*sstT*, Δ*tdcC*)	This work

The low copy number vector pSC is a laboratory stock plasmid and contains the temperature-sensitive lambda-repressor cItS857 gene and the lambda PR and PL promoters. The expression plasmid pSC-08 (pSC-PR-*serB*-PL-*serA*^*fr*^-PR-*serC*-PL-*pgk* with the p15A origin gene, Additional file 5: Fig. S5) is a laboratory stock plasmid and contains the feedback-insensitive *serA*^*fr*^ (H334A, D346A) gene, the *serC* and *serB* genes, and the glucose metabolism-related gene *pgk*. The plasmids pKD13, pKD46 and pCP20 are helper plasmids and are used for the construction of knockout mutants.

In this study, the plasmids pSC-11, pSC-12, pSC-13 and pSC-14 were derived from the plasmid pSC. All l-serine uptake-related genes, namely, *sdaC*, *cycA*, *sstT* and *tdcC*, were cloned from the chromosomal DNA of *E. coli* W3110. For example, the primers *sdaC*-AvrII and *sdaC*-PvuII (Additional file 4: Table S2) were used to amplify *sdaC* by PCR. Then, the amplified fragments were ligated into the pMD-19 vector through TA cloning. After DNA sequencing, *sdaC* was subcloned into the plasmid pSC under the control of the PR promoter to create the plasmid pSC-11. The plasmids pSC-12, pSC-13 and pSC-14 were obtained by inserting *cycA*, *sstT* and tdcC into the plasmid pSC, respectively, using the same method described above. All constructed plasmids are listed in Table 2.

**Table 2:** Plasmids used in this study

Plasmid	Relevant characteristics	Source
pKD13	*amp* and *kan* markers	[24]
pKD46	*amp* markers, temperature-sensitive	[24]
pCP20	*amp* and *Chl* markers, temperature-sensitive	[24]
pMD-19	PCR cloning vector	Takara
pSC	Low copy number, *kan* marker, p15A replicon, lambda PR and PL promoters	Stock in lab
pSC-08	pSC derivative, carrying *serA*^*fr*^ under PL promoter, *serB* under PR promoter, *serC* under PR promoter and *pgk* under PL promoter	Stock in lab
pSC-11	pSC derivative, carrying *sdaC* under PR promoter	This work
pSC-12	pSC derivative, carrying *cycA* under PR promoter	This work
pSC-13	pSC derivative, carrying *sstT* under PR promoter	This work
pSC-14	pSC derivative, carrying *tdcC* under PR promoter	This work

### Media

LB medium (10 g/L tryptone, 10 g/L NaCl, 5 g/L yeast extract) was used to culture the strains for the serine uptake assays. For l-serine production, minimal M9 medium (6.8 g/L Na_2_HPO_4_, 3 g/L KH2PO_4_, 0.5 g/L NaCl, 1 g/L NH_4_Cl, 0.015 g/L CaCl_2_·2H_2_O, 0.49 g/L MgSO_4_·7H_2_O and 2.8 × 10^−4^ g/L MgSO_4_·7H_2_O) with 2 g/L yeast extract and 9 g/L glucose was used with a shake flask. Fed-batch cultures contained 3 g/L MgSO_4_·7H_2_O, 0.017 g/L CaCl_2_·2H_2_O, 1 g/L NaCl, 5 g/L (NH_4_)_2_SO_4_, 0.07 g/L FeSO_4_·7H_2_O, 0.11 g/L Na-citrate·2H_2_O, 2 g/L yeast extract, 8 g/L glucose and 1.5 mL/L 1000 × mother liquor of a composite additive of trace elements (7 g/L CoCl_2_·6H_2_O, 2.5 g/L CuSO_4_·5H_2_O, 25 g/L H_3_BO_3_, 16 g/L MnCl_2_·4H_2_O, 1.5 g/L Na_2_MoO_4_·2H_2_O, 3 g/L ZnSO_4_·7H_2_O).

### Shake flask and fed-batch fermentation

For shake flask studies, a single clone was first grown in 5 mL of LB for 12–14 h, and then, 5 mL was transferred to 100 mL of M9 medium with 2 g/L yeast extract and 9 g/L glucose and grown in a 500-mL shake flask at 30 °C and 200 rpm. Each culture was induced after 3 h by heating to 38 °C. The shake flask studies were replicated three or more times.

All fed-batch fermentations were conducted in a 5-L bioreactor (Biostat A Plus, Sartorius Stedim, Germany). A single clone was precultured in 50 mL of LB medium and shaken at 33 °C and 200 rpm for 12 to 14 h. The culture was inoculated into 2.5 L of fermentation medium at a 1:20 (v/v) inoculum ratio with an initial temperature of 33 °C. l-serine production was induced at 8 h by heating to 38 °C. The agitation, air supplementation and feed rate were changed to maintain the dissolved oxygen (DO) concentrations above 30% saturation. The pH was controlled at 6.8 using 30% (w/v) NH_3_·H_2_O. The DO-stat feeding strategy was employed to supply exhausted nutrients to the fermenter. The feeding solution contained 40% (w/w) glucose.

### Analysis methods

The cell density was determined from the OD_600_ by using a UV/vis spectrophotometer (DU730, Beckman, Germany) and converted into the cell dry weight (CDW) using a precalibrated conversion factor of 0.5. Glucose was measured offline based on glucose oxidase (GOx) binding to electrode transducers, which determines glucose based on amperometric (anodic) monitoring of liberated hydrogen peroxide, by an SBA sensor machine (Institute of Microbiology, Shandong, China) [25].

For quantification of l-serine, the culture was centrifuged (16,904×*g* for 10 min) and filtered through a 0.22-µm syringe filter to prepare cell-free supernatant. Then, the supernatant was precolumn derived as described in previous research [26]. The samples obtained were used for HPLC analysis with a Shimadzu Separations module connected to a Shimadzu SPD-M20A detector set to 256 nm. The samples were separated on an Agilent Extend C-18 column (250 mm × 4.6 mm, 5 µm) with 0.05 mol/L sodium acetate (pH 6.50 ± 0.05) (mobile phase A) and methanol/acetonitrile/water (20:60:20, v/v/v) (mobile phase B). The following gradient was used at a flow rate of 0.8 mL/min for 25 min: from 0 min to 11 min, 85% solvent A + 15% solvent B; at 11 min, the ratio of solvent B increased to 100%; at 12 min, 0% solvent A + 100% solvent B; from 15 to 17 min, the ratio of solvent B decreased to 15%; and from 17 min to 25 min, 85% solvent A + 15% solvent B.

### l-Serine uptake activity assay

Cells were cultured in 200 mL of LB medium to OD_600_ ~ 2.0 at 37 °C with shaking at 200 rpm, centrifuged and washed twice with Buffer X (7.2 mmol/L K_2_HPO_4_, 2.8 mmol/L KH_2_PO_4_, 100 mmol/L NaCl) followed by centrifugation at 8608×*g* and 4 °C for 3 min. Eventually, the cells were resuspended in 50 mL of Buffer X containing an additional 10 mmol/L l-serine and 20 mmol/L glucose and cultivated at 37 °C for the l-serine uptake assay. Samples were collected every 5 min and centrifuged at 8608×*g* at 4 °C for 2 min. The cell-free culture supernatant was collected and assayed by HPLC. The l-serine uptake activity was defined as the nanomoles of serine taken up per milligram of dry cell weight according to a previous method [27, 28].

### Total RNA isolation, cDNA synthesis and qPCR conditions

First, 500 μL samples in log-phase were collected and immediately frozen in liquid nitrogen. Then, the cells were used for RNA isolation with a UNlQ-10 Column TRIzol Total RNA Isolation Kit (Sangon Biotech, China). The purity of the total extracted RNA was analysed by a NanoDrop spectrophotometer (NanoDrop 2000c, Thermo Scientific, USA), and the integrity was checked by electrophoresis on a 1.5% agarose gel. RNA samples were subjected to cDNA synthesis using a High Capacity cDNA Reverse Transcription Kit (Thermo Scientific, USA).

qPCRs were performed using a StepOnePlus Real-Time PCR System (ABI, Foster, CA, USA). The primer pairs used for amplifying the genes are shown in Additional file 6: Table S3. The PCR mixture consisted of 10 μL of 2 × SybrGreen qPCR Master Mix, 20 µM forward and reverse primers, 20 ng of cDNA template and nuclease-free water in a total volume of 20 μL. Thermocycling was performed using the following conditions: 3 min at 95 °C followed by 40 cycles alternating between 5 s at 95 °C and 30 s at 60 °C. Melting curve analysis (60–95 °C) was routinely performed after 45 cycles to verify primer specificity. The 2^−ΔΔCt^ method was used to calculate the relative expression level of the target genes [29].

## Supplementary information

**Additional file 1.** HPLC chromatograms of L-serine standard solution and fermentation broth.

**Additional file 2.** L-serine concentration in the L-serine uptake activity assay.

**Additional file 3.** Fermentation parameters of *E. coli* ES-1/pSC-08, ES-13/pSC-08, ES-134/pSC-08 and ES-1234/pSC-08.

**Additional file 4.** Primers used for deletion and overexpression.

**Additional file 5.** Structure of the plasmid pSC-08.

**Additional file 6.** Primers used for RT-qPCR.

## Data Availability

All data generated and analysed during this study are included in this published article and its additional files.

## Abbreviations

Fr: Feedback-inhibition resistance
SHMT: l-serine hydroxymethyltransferase
GABA: Aminobutyric acid
LB: Luria–Bertani
GOx: Glucose oxidase
RT-qPCR: Real-time quantitative reverse‐transcription PCR
DO: Dissolved oxygen
CDW: Cell dry weight
HPLC: High-performance liquid chromatography
