# Supplementary material for: Improving l-serine formation by Escherichia coli by reduced uptake of produced l-serine
Source: Microb Cell Fact. 2020 Mar 14;19:66. doi: 10.1186/s12934-020-01323-2 (PMC7071685; doi:10.1186/s12934-020-01323-2)
Supplement: Supplementary file 1 — Additional file 1. HPLC chromatograms of L-serine standard solution and fermentation broth. [file 12934_2020_1323_MOESM1_ESM.docx]

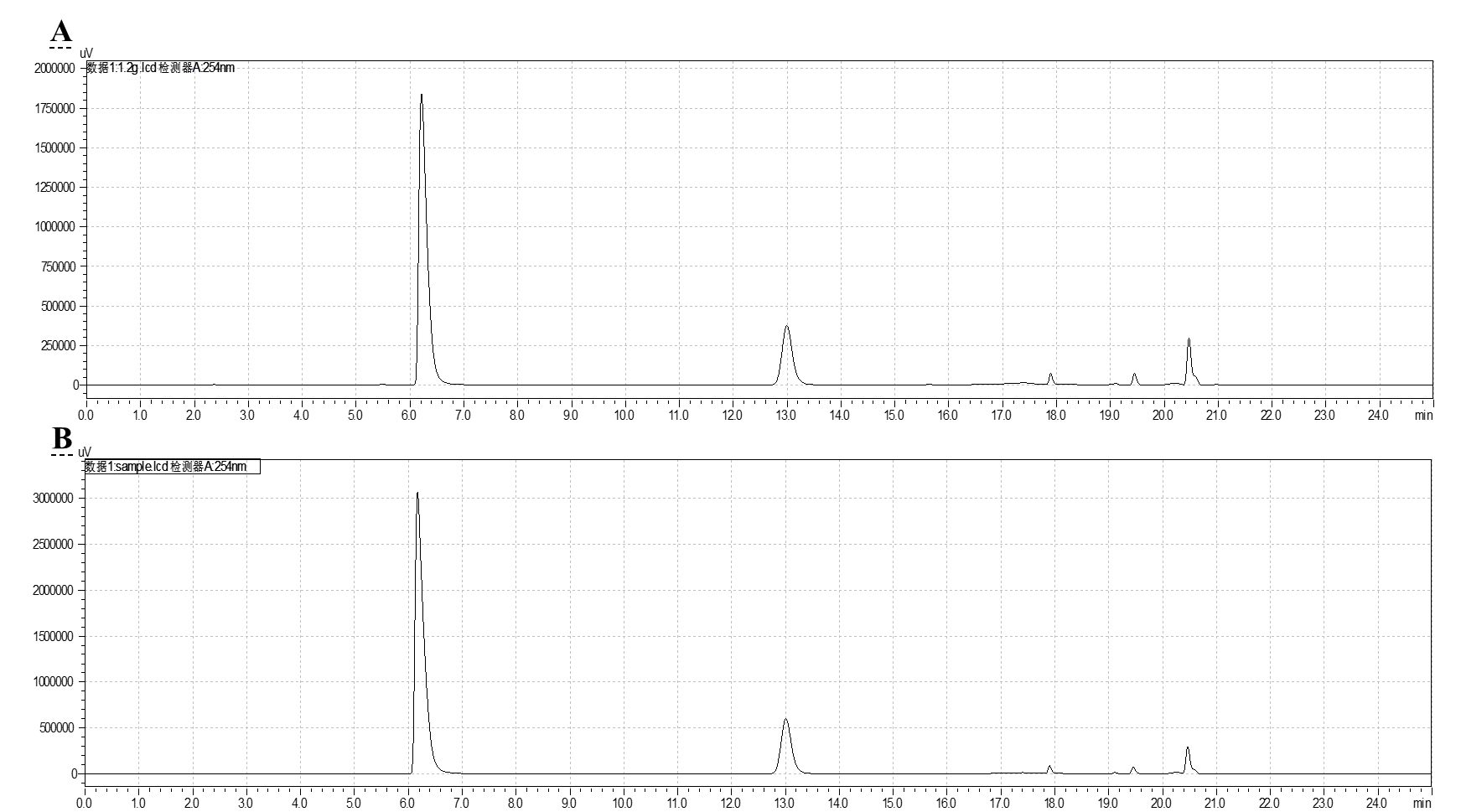
**Fig S1 HPLC chromatograms of L-serine standard solution and fermentation broth**

1. HPLC chromatogram of L-serine standard solution (1.2 g/L).
2. HPLC chromatogram of *E. coli* ES-134/pSC-08 fed-batch fermentation broth at 36 h.
