# Supplementary material for: Improving l-serine formation by Escherichia coli by reduced uptake of produced l-serine
Source: Microb Cell Fact. 2020 Mar 14;19:66. doi: 10.1186/s12934-020-01323-2 (PMC7071685; doi:10.1186/s12934-020-01323-2)
Supplement: Supplementary file 2 — Additional file 2. L-serine concentration in the L-serine uptake activity assay. [file 12934_2020_1323_MOESM2_ESM.docx]

## Fig. S2 L-serine [concentration](file:///D:\Program%20Files%20(x86)\Dict\7.5.2.0\resultui\dict\?keyword=concentration) in the L-serine uptake activity assay of *E. coli* ES and mutants overexpressing the L-serine uptake genes

The data represent the means ± SDs from three measurements. ES represents the parental strain.

# Fig. S3 L-serine [concentration](file:///D:\Program%20Files%20(x86)\Dict\7.5.2.0\resultui\dict\?keyword=concentration) in the L-serine uptake activity assay of *E. coli* ES and single-gene knockout mutants.

The data represent the means ± SDs from three measurements. ES represents the parental strain. ES-1: ES Δ*sdaC*; ES-2: ES Δ*cycA*; ES-3: ES Δ*sstT*; ES-4: ES Δ*tdcC.*

# Fig. S4 L-serine [concentration](file:///D:\Program%20Files%20(x86)\Dict\7.5.2.0\resultui\dict\?keyword=concentration) in the L-serine uptake activity assay of *E. coli* ES and multigene deletion mutants

The data represent the means ± SDs from three measurements. ES represents the parental strain. ES-1: ES Δ*sdaC*; ES-12: ESΔ*sdaC*Δ*cycA*; ES-13: ESΔ*sdaC*Δ*sstT;* ES-14: ESΔ*sdaC*Δ*tdcC*; ES-123: ES Δ*sdaC*Δ*cycA*Δ*sstT*, ES-124: ESΔ*sdaC*Δ*cycA*Δ*tdcC*; ES-134: ESΔ*sdaC*Δ*sstT*Δ*tdcC;* ES-1234: ESΔ*sdaC*Δ*cycA*Δ*sstT*Δ*tdcC*
