# Supplementary material for: Improving l-serine formation by Escherichia coli by reduced uptake of produced l-serine
Source: Microb Cell Fact. 2020 Mar 14;19:66. doi: 10.1186/s12934-020-01323-2 (PMC7071685; doi:10.1186/s12934-020-01323-2)
Supplement: Supplementary file 3 — Additional file 3. Fermentation parameters of E. coli ES-1/pSC-08, ES-13/pSC-08, ES-134/pSC-08 and ES-1234/pSC-08. [file 12934_2020_1323_MOESM3_ESM.docx]

# Table S1 Fermentation parameters of *E. coli* ES-1/pSC-08, ES-13/pSC-08, ES-134/pSC-08 and ES-1234/pSC-08

| Parameter | ES-1/pSC-08 | ES-13/pSC-08 | ES-134/pSC-08 | ES-1234/pSC-08 |
| --- | --- | --- | --- | --- |
| Maximum OD_600_ | 43.3±0.7 | 40.3±1.5 | 40.3±1.3 | 30.3±1.7 |
| Maximum L-serine titer (g/L) | 23.8±0.6 | 29.6±0.7 | 34.8±1.0 | 26.3±0.5 |
| Maximum productivity (mg L-serine/g CDW h^-1^) | 35.5±1.9 | 46.5±1.6 | 56.1±0.2 | 57.1±2.0 |
| Glucose consumption (g/L) | 95±2 | 97±3 | 109±6 | 90±6 |
| L-Serine yield on glucose (g L-serine/g glucose) | 0.25±0.002 | 0.31±0.017 | 0.32±0.016 | 0.29±0.014 |

The values of fermentation parameters were determined during the effective L-serine production time (12 h- 36 h).

The values represent the means ± SDs from three measurements.
