# Supplementary material for: Improving l-serine formation by Escherichia coli by reduced uptake of produced l-serine
Source: Microb Cell Fact. 2020 Mar 14;19:66. doi: 10.1186/s12934-020-01323-2 (PMC7071685; doi:10.1186/s12934-020-01323-2)
Supplement: Supplementary file 4 — Additional file 4. Primers used for deletion and overexpression. [file 12934_2020_1323_MOESM4_ESM.docx]

## Table S2. Primers used for deletion and overexpression.

| **Primer** | **Sequence (5ʹ–3ʹ) ^a, b, c^** |
| --- | --- |
| Knockout primers | |
| *sdaC*-p1 | GGCTGAACTGGCTAAAAGCTGAATTATTTGCATTCCTCCAGGAGAAATAGATTCCGGGGATCCGTCGACC |
| *sdaC*-p2 | ACATCGCGTTAAAACGGAGGAAGCGCCGCCCGAAAGCGGCGCGAAGGACTGTAGGCTGGAGCTGCTTCG |
| *cycA*-p1 | GGCTGAAACGGTGAGTGGTTTCGCGTACCACCATTGCATCAACATCCAGCATTCCGGGGATCCGTCGACC |
| *cycA*-p2 | TATAGCCCTGCCCCTGCCATACCGGCATGATGTGCGCGGCATCGCCCGCCTGTAGGCTGGAGCTGCTTCG |
| *sstT*-p1 | TCGACAGAACGCACCAGGGATGTGCGACAACACAATGAAAGGATCGAAAAATTCCGGGGATCCGTCGACC |
| *sstT*-p2 | GTTTAAAGTTGAGAAAACCCCTTCCGCCGTAGACGAAAGGGGTTAAACAATGTAGGCTGGAGCTGCTTCG |
| *tdcC*-p1 | CCAAAACAACCGGAAATTCATTCATCTCTTTTCTCATCCTGAGTTACGGAATTCCGGGGATCCGTCGACC |
| *tdcC*-p2 | CTCAAATCACCGGTTTCGTTGACGCTTAATTAATTCGTTGAGGATAGGATTGTAGGCTGGAGCTGCTTCG |
| Verification primers | |
| *sdaC*-v1 | GAAGTCGGTATTCGCGCCATTGAAGAG |
| *sdaC*-v2 | GATCGGCTGCTGAACTGTACGGATAAG |
| *cycA*-v1 | ATAGCACCGAATCTGGCCTGCAATTC |
| *cycA*-v2 | CTGGATGTTGAGGTCATTGAAGCTGAAC |
| *sstT*-v1 | CTGGTGTTTATCGGTATCAAGATGCTG |
| *sstT*-v2 | CTGGAAGAATTTATCGACACCATCGTTGAG |
| *tdcC*-v1 | GTCATCTTCCGCCAGATTCAGCAGC |
| *tdcC*-v2 | CAAAGTAGCGGCAACGTGCGACTAC |
| K1 | CAGTCATAGCCGAATAGCCT |
| K2 | CGGTGCCCTGAATGAACTGC |
| Overexpression primers | |
| *sdaC-**Avr*Ⅱ | cctaggCGTTAAAACGGAGGAAGC |
| *sdaC*-*Pvu*Ⅱ | gatatcGGCTGAACTGGCTAAAAGCTG |
| *cycA*- *Avr*Ⅱ | cctaggAGCTGGATGGCATTGC |
| *cycA*- *Pvu*Ⅱ | gatatcGAGCCTGAACAACACAGACAG |
| *sstT*- *Avr*Ⅱ | cctaggACAGAACGCACCAGGGATG |
| *sstT*- *Pvu*Ⅱ | cagctgTTGAGAAAACCCCTTCCG |
| *tdcC*- *Avr*Ⅱ | cctaggTCAAATCACCGGTTTCGTTG |
| *tdcC*- *Pvu*Ⅱ | gatatcCAACCGGAAATTCATTCATCTC |

^a^ Underlined bases represent homologous arms of each serine uptake gene.

^b^ Primer pair *gene*-v1 and K1 and primer pair *gene*-v2 and K2 were used for colony PCR verification.

^c^ Sequence in lowercase letters represents restriction enzyme cleavage sites.
