# Supplementary material for: Improving l-serine formation by Escherichia coli by reduced uptake of produced l-serine
Source: Microb Cell Fact. 2020 Mar 14;19:66. doi: 10.1186/s12934-020-01323-2 (PMC7071685; doi:10.1186/s12934-020-01323-2)
Supplement: Supplementary file 5 — Additional file 5. Structure of the plasmid pSC-08. [file 12934_2020_1323_MOESM5_ESM.docx]

Fig. S5 Structure of the plasmid pSC-08

**

Abbreviations: kan, kanamycin resistance gene; *cIts*, temperature-sensitive variant of the phage λ repressor; PR and PL are promoters; *serA-fr*, 3-phosphogylcerate dehydrogenase gene (H334A, D346A) in *E. coli*; *serB*, 3-phosphoserine phosphatase gene in *E. coli*; *serC*, 3-phosphoserine aminotransferase gene in *E. coli*; *pgk*, 3-phosphoglycerate kinase gene in *E. coli.*
