# Supplementary material for: Improving l-serine formation by Escherichia coli by reduced uptake of produced l-serine
Source: Microb Cell Fact. 2020 Mar 14;19:66. doi: 10.1186/s12934-020-01323-2 (PMC7071685; doi:10.1186/s12934-020-01323-2)
Supplement: Supplementary file 6 — Additional file 6. Primers used for RT-qPCR. [file 12934_2020_1323_MOESM6_ESM.docx]

## Table S3. Primers used for the RT-qPCR.

| **Primer** | **Sequence (5ʹ–3ʹ)** |
| --- | --- |
| *sdaC*-F | GGATTGTTGCCACCCTGAA |
| *sdaC*-R | TGTGACCGCTGTACTTACGC |
| *cycA*-F | GCGTGGTGATGTTGTATGTGA |
| *cycA*-R | CACAAGGTACGAGCAAAGGAT |
| *sstT*-F | TCCTCTATCTACTGGGCACCTT |
| *sstT*-R | TGACGGCGGCGAAATAT |
| *tdcC*-F | GCAGACCAATCACGGTAACAA |
| *tdcC*-R | TCGCTGGGTAAACTGAACACTA |
| *16S*-F | ACTCCTACGGGAGGCAGCAG |
| *16S*-R | ATTACCGCGGCTGCTGG |
